# p53 codon 72 polymorphism association with head and neck squamous cell carcinoma

**DOI:** 10.1590/S1808-86942010000300008

**Published:** 2015-10-20

**Authors:** Zahra Mojtahedi, Seyed Basir Hashemi, Bijan Khademi, Morteza Karimi, Mohammad Reza Haghshenas, Mohammad Javad Fattahi, Abbas Ghaderi

**Affiliations:** 1 Dr. (Assistant Professor, Shiraz Institute for Cancer Research, Shiraz University of Medical Sciences, Shiraz, Iran.); 2 Dr. (Associate Professor, Department of Ear Nose and Throat Surgery, Khalili Hospital, Shiraz University of Medical Sciences, Shiraz, Iran.); 3 Professor. (Professor, Department of Ear Nose and Throat Surgery, Khalili Hospital, Shiraz University of Medical Sciences, Shiraz, Iran.); 4 Dr. (Dr, Department of Ear Nose and Throat Surgery, Khalili Hospital, Shiraz University of Medical Sciences, Shiraz, Iran.); 5 Mr. (MSc, Shiraz Institute for Cancer Research, Shiraz University of Medical Sciences, Shiraz, Iran.); 6 MR. (MSc, Shiraz Institute for Cancer Research, Shiraz University of Medical Sciences, Shiraz, Iran.); 7 Professor. (Director of Shiraz Institute for Cancer Research, Shiraz University of Medical Sciences, Shiraz, Iran.)

**Keywords:** genes, head and neck neoplasms, prognosis, tumor suppressor

## Abstract

**p53** tumoral suppressor gene harbors a functional polymorphism which codes either arginine (Arg) or proline (Pro) in the protein p53 of codon 72. Such polymorphism has been associated with the development or prognosis of head and neck squamous cell carcinoma (HNSCC).

**Aim:** we assessed codon 72 p53 allelic frequencies and genotypes in HNSCC Iranian patients.

**Study design:** Case Study.

**Materials and Methods:** a total of 132 HNSCC patients and 123 healthy controls were genotyped. DNA source was from mononuclear cells of the peripheral blood. DNA amplification was done by means of the allele-specific polymerase chain reaction.

**Results:** genotypes and allele distribution were not significantly different between patients and controls. Moreover, no statistically significant association was found between the 72 and p53 codon tumor location, gender or age at the time of diagnosis. However, the Pro/Pro genotype was significantly increase in stage IV patients (30.8%) when compared to stages I-III of the disease (11.1%) (p=0.03), and a significantly higher percentage of patients with the Pro allele had and a risk increase in stage IV disease (OR=2.2, 95% CI=1.2-4.2, p=0.01).

**Conclusion:** data revealed that the p53 polymorphism do not impact the risk of HNSCC in Iranians, nonetheless, it can affect tumor progression to a higher tumor stage.

## INTRODUCTION

Head and neck carcinomas are a group of malignant tumors originating form the upper aerodigestive tract including the oral cavity, pharynx, and larynx. More than 90% of head and neck carcinomas are squamous cell carcinoma, and arise from the epithelial tissues of these regions^1^.

The incidence of head and neck squamous cell carcinoma (HNSCC) shows a wide range of geographical variation, with 500 000 new cases worldwide, and is more frequent in developing countries. Tobacco and alcohol consumption are recognized as the most common environmental carcinogens for HNSCC. However, environmental factors can be remarkably influenced by the host genetic background^1^,^2^.

The tumor suppressor p53 is a sequence-specific DNA-binding transcription factor that acts as the major cellular gatekeeper for growth and division. Normal cells express a low concentration of this nuclear protein in an inactive form, but in response to a wide variety of cellular stresses such as DNA damage, hypoxia, or the depletion of ribonucleoside triphosphate pools, p53 is activated and overexpressed. After activation, p53 mediates either 1) cell cycle arrest to allow DNA repair, or 2) apoptosis to remove the irreparably damaged cells^3^.

The p53 gene, located on chromosome band 17p13.1, is susceptible to somatic mutation that is estimated to occur in 50% of all human cancers^3^. The majority of these mutations cause dramatic defects in p53 function. In contrast, only a small fraction of inherited polymorphisms are expected to cause detectable perturbation of p53 function^3^,^4^. Codon 72 polymorphism in exon 4 is the most commonly studied p53 polymorphism that encodes either arginine (Arg) or proline (Pro). These variants are suggested to alter the structure and function of the p53 protein. The Pro allele, for instance, induces apoptosis with slower kinetics and represses transformation less efficiently compared to the Arg allele^5^.

Attempts to define the associations of p53 codon 72 polymorphism with cancer susceptibility or progression have yielded inconsistent results in different ethnic backgrounds and different types of cancer, including HNSCC^6^, ^7^, ^8^, ^9^, ^10^, ^11^. Because there are no existing data for Iranian patients with HNSCC, we investigated the association between HNSCC susceptibility and p53 codon 72 polymorphism in a case-control study. In addition, the association between this polymorphism and gender, tumor location, age and disease stage at diagnosis was evaluated.

## PATIENTS AND METHODS

### Patients

All participants were informed that blood samples would be used for genotyping, and their consent was obtained. The Ethics Committee of Shiraz University of Medical Sciences approved the study. Shiraz University of Medical Sciences is a tertiary institution financially supported by the Ministry of Health and Medical Education of Iran.

A total of 132 unrelated patients with HNSCC (97 men and 35 women; mean age 56.6±14.1 years) were recruited from a Shiraz University of Medical Sciences hospital (Khalili hospital) in Shiraz, Iran. The diagnosis of SCC was confirmed histopathologically for all patients. Patients' characteristics including gender, tumor location, age at diagnosis, metastasis, and TNM clinical staging were obtained from their medical records. The tumor was located in the oral cavity in 52 cases (39.4%), in the pharynx in 15 cases (11.4%) and in the larynx in 65 cases (49.2%). The mean age at onset of the disease was 55.9±13.1 years, ranging from 19 to 83 years. Disease stage was determined according to the TNM classification. Control group comprised

123 healthy volunteers (93 men and 30 women; mean age 58.7±12.4 years) residing in the same region as patients. Control participants did not have any type of cancer or first-degree family history of cancer. The native language of all participants was Persian.

### DNA preparation and polymerase chain reaction

Blood samples were collected in EDTA-containing tubes and genomic DNA was extracted from white blood cells with salting out method 12. Briefly, whole blood cells were first treated with a red blood cell lysis solution (155 mM NH4CL and 10 mM NaHCO3) and separated from white cells by centrifugation. Thereafter white cells were lysed in a nuclei lysis buffer (10 mM Tris-HCl, 400 mM NaCl and 2 mM Na2EDTA, pH 8.2). The cell lysate was digested overnight at 37°C with 10% SDS and a proteinase K solution (1 mg proteinase K in 1% SDS and 2 mM Na2EDTA). Saturated NaCl (approximately 6M) was then added to tubes and shaken vigorously, followed by centrifugation. The precipitated protein pellet was left, and the supernatant containing the DNA was transferred to another tube and mixed with ethanol. The tubes inverted several times until the DNA precipitated. The precipitated DNA strands were transferred to a 1.5 ml microcentrifuge tube containing TE buffer (10 mM Tris-HCl, 0.2 mM Na2EDTA, pH 7.5). The DNA was allowed to dissolve 2 hours at 37°C before quantitating.

The amplification of DNA was done by an allele-specific polymerase chain reaction (PCR) method as previously described^13^. Two sets of primers were used to detect the Arg and the Pro alleles. The nucleotide sequences for p53-Arg specific primers were 5′- TCC CCC TTG CCG TCC CAA-3′ (forward primer) and 5′-CTG GTG CAG GGG CCA CGC-3′ (reverse primer). The primer sequences for the amplification of the Pro allele were 5′-GCC AGA GGT TGC TCC CCC-3′ (forward primer), and 5′-CGT GCA AGT CAC AGA CTT-3′ (reverse primer).

Each sample was amplified by each set of primers in separate tubes containing 0.3 mmol/L dNTPs, 1.5 mmol/L MgCl2, 2 U Taq polymerase, and 1 x buffer (20 mmol/L Tris-HCl, pH 8.4, and 50 mmol/L KCl) (total volume of 25 μL). Amplification was performed at 35 cycles with a touch-down program: denaturation at 94°C for 30 seconds, annealing at 68-62°C for 10 cycles and 62-58°C for the remaining 25 cycles, with extension at 72°C for 30 seconds in each cycle. PCR products were separated by electrophoresis on 2% agarose gel (invitrogen, UK) and stained with ethidium bromide. The PCR product was 177 bp for the Pro allele and 141 bp for the Arg allele.

### Statistical analysis

Genotype frequencies were tested for the Hardy-Weinberg equilibrium. Fit to the equilibrium was checked with the chi-squared test. The data were analyzed with SPSS software (version 11.5.0; SPSS, Chicago, IL, USA). Differences in the mean age of patients with different p53 codon 72 genotypes were calculated with one-way analysis of variance (ANOVA). Other analyses were done with Pearsons chi-squared test. The differences were considered statistically significant at a P value less than 0.05. Odds ratios (OR) and 95% confidence intervals (CI) were calculated by the Mantel- Haenszel method to estimate the relative risk associated with alleles.

## RESULTS

Genotype frequencies did not differ significantly from those expected according to the Hardy-Weinberg equilibrium in patients or control participants. The frequencies of Arg/Arg, Arg/Pro, and Pro/Pro genotypes were 37.1%, 47.7%, and 15.2% in patients with HNSCC, and 35.8%, 51.2%, and 13.0% in controls, respectively (Table 1). Arg allele frequency was 61.0%, and 61.4% in patients and controls, respectively. Pro allele frequency was 39.0% in patients and 38.6% in controls. There was no statistically significant difference in genotype frequency between the patient group and the control group (p=0.82). Allele frequencies did not differ significantly between the two groups (p=0.92).

**Table 1:** Genotype and allele frequencies of the p53 codon 72 polymorphism in 132 patients with head and neck squamous cell carcinoma and 123 healthy controls

	Patients	Controls
	n (%)	n (%)
Alleles		
Arginine	161 (61.0)	151 (61.4)
Proline	103 (39.0)	95 (38.6)
Genotypes		
Arginine/Arginine	49 (37.1)	44 (35.8)
Arginine/Proline	63 (47.7)	63 (51.2)
Proline/Proline	20 (15.2)	16 (13.0)

No statistically significant difference was found between patients and controls in any of the comparisons as calculated with the chi-squared test in 2 × 3 or 2 × 2 tables.

The associations between p53 codon 72 genotypes and gender, age at diagnosis, tumor location, metastasis and TNM clinical stage are summarized in Table 2. The only significant association with the distribution of different genotypes was found for tumor stage (p=0.03). The Pro/ Pro genotype was more frequently found in patients with stage IV than patients with stage (I-III) disease (p=0.032). Although the frequency of this genotype was increased in patients with metastasis, the increase did not reach statistical significance (p=0.07).

**Table 2:** Association between clinicopathological characteristics of 132 patients with head and neck squamous cell carcinoma and p53 codon 72 genotypes

Characteristics	Genotypes			p value^a^
	Arg/Arg	Arg/Pro	Pro/Pro	
Mean age ±SD (years)	57,4±12,5	55±14,7	54,6±9,8	0,63
Gender, n (%)				0,98
Men	36 (37,1)	46 (47,4)	15 (15,5)	
Women	13 (37,1)	17 (48,6)	5 (14,3)	
Tumor location				0,27
Oral cavity	16 (30,8)	30 (57,7)	6 (11,5)	
Pharynx	4 (26,7)	8 (53,3)	3 (20,0)	
Larynx	29 (44,6)	25 (38,5)	11 (16,9)	
Metastasis^b^				0,07
Negative	37 (38,9)	46 (48,5)	12 (12,6)	
Positive	3 (30,0)	3 (30,0)	4 (40,0)	
Tumor stage^b^				0,03
I-III	35 (43,2)	37 (45,7)	9 (11,1)	
IV	6 (23,1)	12 (46,1)	8 (30,8)	

a The p value of the differences in mean age was calculated by the one-way ANOVA; other p values were calculated with the chi-squared test.

b The sum does not equal 100% because of a few missing values.

As shown in Table 3, the frequency of the Pro allele and the Arg allele were 28 (53.8%) and 24 (46.2) in patients with stage IV, and 55 (34.0%) and 107 (66.9) in patients with stage I-III disease; this difference was also significant (p=0.01), and carriers of the Pro allele had a 2.2-fold higher risk of being diagnosed with stage IV (95% CI = 1.2-4.2).

**Table 3:** Allele frequency of the p53 codon 72 polymorphism according to tumor stage

Allele	Stage I-III	Stage IV	p value	OR (95% CI)^a^
Arg	107 (66.0)	24 (46.2)	0.01	2.2 (1.2-4.2)
Pro	55 (34.0)	28 (53.8)		

a Odds ratio with 95% confidence interval calculated with the Mantel-Haenszel method.

## DISCUSSION

The p53 tumor suppressor gene is involved in DNA repair, the cell cycle, gene transcription, and apoptosis^3^,^4^; it therefore represents an attractive candidate for attempts to explain HNSCC susceptibility and inter-individual variations in prognosis. The p53 codon 72 Arg/Pro polymorphism is a functional polymorphism whose genotype distribution is significantly different among ethnic groups. It has been shown that the Pro allele frequency varies from 17% in Swedish Saamis to 63% in African Blacks (Nigerians), and the proportion of Arg allele carriers is higher in populations who live farther away from the Earths equator^14^. The association of this polymorphism with cancers, including HNSCC, in different ethnic backgrounds is the subject of controversy^6^, ^7^, ^8^, ^9^, ^10^, ^11^. For instance, the p53 codon 72 polymorphism was significantly associated with HNSCC in Italian8, but not in Indian^9^ populations. The high variability of the p53 codon 72 Arg/Pro polymorphism among populations has frequently been cited to explain differences observed in codon 72 in persons with cancer from different ethnic backgrounds^7^. It seems that the correlation of the codon 72 with cancer should be determined in each population.

We compared the allele and genotype frequencies of the p53 codon 72 Arg/Pro polymorphism in HNSCC patients and healthy individuals in a southern Iranian population. This is, to our knowledge, the first attempt to identify the associations of this polymorphism with clinical and prognostic features in an Iranian population. We found no significant differences in genotype or allele frequency between patients and healthy control participants. However, the Pro/Pro genotype and the Pro allele, associated with slower kinetics of apoptosis and less efficient suppression of transformation than the Arg allele^5^, were significantly increased in patients with a more advanced disease. According to our results, one can argue that the effect of the Pro allele may be more pronounced in cancer progression than in cancer formation. Although the Pro and the Arg alleles have some differences in their abilities for apoptosis induction and transformation suppression, they do not differ in sequence-specific DNA binding activities^5^. Intriguingly, p53 mutations that are found in 50-55% of all human cancers with an important role in cancer formation strongly select for p53 proteins with defects for binding to DNA^3^. In agreement with our results, Tu et al. in a Taiwanese population found no association between p53 codon 72 polymorphism and susceptibility to oral squamous cell carcinoma, but found that the p53 codon 72 polymorphism was associated with the disease stage, and with overall and disease-free survival of irradiated patients^10^.

In our study population, distribution of the p53 codon 72 polymorphism did not differ significantly among patients according to the sex, age at diagnosis or tumor location. The reported associations of HNSCC characteristics with this polymorphism also vary widely in the literature^10^,^11^. For example, while the Pro allele was associated with an early age of HNSCC onset in non-Hispanic whites^11^, no association was found between p53 codon 72 polymorphism and the age of the disease onset in Taiwanese patients^10^. The p53 pathway is composed of many genes and wide varieties of cellular stress signals that activate p53 result in distinct genes being transcribed^3^. Differences in p53 interacting genes, and also differences in stress signals among various populations may be other explanations for the diverse results.

## CONCLUSION

In conclusion, we detected a significant positive association between the Pro allele of the p53 codon 72 and tumor stage, an important prognostic factor in HNSCC. However, we found no significant association between this polymorphism and risk of HNSCC in Iranian patients. More data from a larger number of patients are needed to determine the possible minor association between the p53 codon 72 Arg/Pro polymorphism and HNSCC risk.

## ACKNOWLEDGMENTS

This work was funded by a grant from Shiraz University of Medical Sciences (Grant No. 83-2102) and by the Shiraz Institute for Cancer Research (Grant No. ICR-82-93). We thank K. Shashok (AuthorAID in the Eastern Mediterranean) for help with improving the use of English in manuscript.
